# Developments and Applications of Electrogenerated Chemiluminescence Sensors Based on Micro- and Nanomaterials

**DOI:** 10.3390/s8095942

**Published:** 2008-09-25

**Authors:** Sandra G. Hazelton, Xingwang Zheng, Julia Xiaojun Zhao, David T. Pierce

**Affiliations:** 1 Department of Chemistry, University of North Dakota, Grand Forks, ND 58202, USA; 2 School of Chemistry and Materials Sciences, Shaanxi Normal University, Xi'an, 710062, P.R. China

**Keywords:** Electrogenerated chemiluminescence, Sensors, Nanomaterials, Fluorescence

## Abstract

A variety of recent developments and applications of electrogenerated chemiluminescence (ECL) for sensors are described. While tris(2,2′-bipyridyl)-ruthenium(II) and luminol have dominated and continue to pervade the field of ECL-based sensors, recent work has focused on use of these lumophores with micro- and nanomaterials. It has also extended to inherently luminescent nanomaterials, such as quantum dots. Sensor configurations including microelectrode arrays and microfluidics are reviewed and, with the recent trend toward increased use of nanomaterials, special attention has been given to sensors which include thin films, nanoparticles and nanotubes. Applications of ECL labels and examples of label-free sensing that incorporate nanomaterials are also discussed.

## 1. Introduction

Electrogenerated chemiluminescence (ECL) is a well-studied phenomenon that bridges the traditional fields of analytical electrochemistry and luminescence spectroscopy. Its basic principles have been well described [1, 2]. Some traits of ELC sensors are common to other luminescent probes (e.g., fluorescence), including high sensitivity and broad linear response. However, unlike fluorescence-based methods, no light source is required in ECL. This difference effectively frees the ECL method from scattered-light interferences [1] and provides unique avenues for sensing, especially in combination with the wide variety of recently developed micro- and nanomaterials that can be used as supports and platforms. Thus, traditional modes of ECL analysis have garnered renewed attention when used in combination with nanotechnology and a great deal of recent work is focused on the development of chemical and biological ECL sensors. In general, these sensors demonstrate remarkably improved characteristics (e.g., sensitivity, detection limits, etc.) for the determination of trace analytes.

ECL can be produced by several distinct approaches. To illustrate the principles behind two common approaches, the most commonly applied lumophore, the ruthenium(II) tris(2,2′-bipyridine) (bpy) complex, [Ru(bpy)_3_]^2+^, will be used. In one approach, an anodic to cathodic potential pulse is applied to an electrode immersed in an electrolyte solution containing [Ru(bpy)_3_]^2+^. Sequential oxidation and reduction of the Ru(II) complex yields [Ru(bpy)_3_]^3+^ and [Ru(bpy)3]^+^ together in the electrode diffusion layer (Equations 1 and 2). These species undergo a highly energetic and favored annihilation reaction, which occasionally leaves one of the resulting Ru(II) complexes in an excited electronic state. This [Ru(bpy)_3_]^2+^* specie (Equation 3) emits light upon relaxation (Equation 4).

(1) $${\left[\text{Ru}{\left(\text{bpy}\right)}_{3}\right]}^{2+}\to {\left[\text{Ru}{\left(\text{bpy}\right)}_{3}\right]}^{3+}$$

(2) $${\left[\text{Ru}{\left(\text{bpy}\right)}_{3}\right]}^{2+}+{\text{e}}^{-}\to {\left[\text{Ru}{\left(\text{bpy}\right)}_{3}\right]}^{+}$$

(3) $${\left[\text{Ru}{\left(\text{bpy}\right)}_{3}\right]}^{3+}+{\left[\text{Ru}{\left(\text{bpy}\right)}_{3}\right]}^{+}\to {\left[\text{Ru}{\left(\text{bpy}\right)}_{3}\right]}^{2+}+{\left[\text{Ru}{\left(\text{bpy}\right)}_{3}\right]}^{2+*}$$

(4) $${\left[\text{Ru}{\left(\text{bpy}\right)}_{3}\right]}^{2+*}\to {\left[\text{Ru}{\left(\text{bpy}\right)}_{3}\right]}^{2+}+hv$$

A second approach for producing ECL involves the participation of a co-reactant. With the [Ru(bpy)_3_]^2+^ lumophore, this co-reactant is typically a tertiary amine or oxalate. Tripropylamine (TPA) generally gives the highest intensity ECL in the presence of [Ru(bpy)_3_]^2+^. The mechanism for this reaction is complex, containing multiple pathways to ECL as shown in Equations 5-8. In the final step, an electrogenerated TPA radical reacts with [Ru(bpy)_3_]^+^ in a highly energetic and favored process to produce the excited-state species, [Ru(bpy)_3_]^2+^*. With co-reactants, not only is the ECL signal often greater than with the annihilation method, but less positive electrode potentials are required since [Ru(bpy)_3_]^2+^ need not be electrochemically oxidized [1].

(5) $$\text{TPA}\to {\text{TPA}}^{•+}+{\text{e}}^{-}$$

(6) $${\text{TPA}}^{•+}\to {\text{TPA}}^{•}+{\text{H}}^{+}$$

(7) $${\text{TPA}}^{•}+{\left[\text{Ru}{\left(\text{bpy}\right)}_{3}\right]}^{2+}\to {\left[\text{Ru}{\left(\text{bpy}\right)}_{3}\right]}^{+}+{\text{TPA}}^{+}$$

(8) $${\left[\text{Ru}(\text{bpy})3\right]}^{+}+{\text{TPA}}^{•+}\to {\left[\text{Ru}{\left(\text{bpy}\right)}_{3}\right]}^{2+*}+\text{products}$$

**Figure 1.**
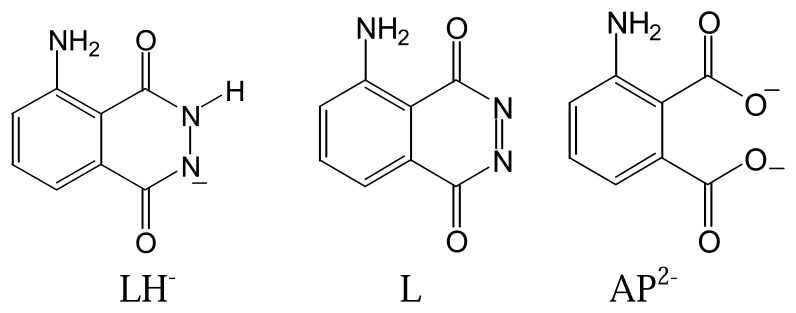
Structures of luminol (LH-), its oxidized form (L) and lumophoric product (AP^2-^).

While the approaches described above are probably the most commonly used to generate ECL in sensing applications, the pathways for producing ECL can be different with different lumophores. For instance, luminol is another common ECL reagent that is used in the determination of or in conjunction with hydrogen peroxide. Its structure is shown in Figure 1.

Luminol's electrochemically oxidized product (L) can react with H_2_O_2_ to form the excited species (AP^2-^*) to give fluorescence signals. The entire ECL process for luminol is described in Equations 9–11 [3].

(9) $${\text{LH}}^{-}\to \text{L}+{\text{H}}^{+}+2{\text{e}}^{-}$$

(10) $$\text{L}+{\text{H}}_{2}{\text{O}}_{2}\to {\text{AP}}^{2-*}+{\text{N}}_{2}+2{\text{H}}^{+}$$

(11) $${\text{AP}}^{2-*}\to {\text{AP}}^{2-}+hv$$

Based on the ECL principle, a wide variety of sensors have been developed. In this article we focus on various materials used for fabricating ECL sensors, including thin films, electrode arrays, microfluidics, and nanomaterials. Particular attention is paid to the use of micro- and nano- sized materials in recent ECL sensors. Then, we summarize two major types of applications of ECL in sensors. In one type the ECL material is used as labeling reagent, and in the other type the ECL sensors is label-free. Finally, the future direction of ECL sensors is briefly discussed.

## 2. Sensing Methods and Materials

A number of materials have been employed for fabrication of ECL sensors. To better evaluate and compare theses sensors we have assembled works that have used a common ECL assay system – TPA detection with a [Ru(bpy)_3_]^2+^ – in order to assess different sensor characteristics. Table 1 lists recent typical ECL sensors and their performance. The lowest detection limit of ECL sensor can reach to 10^-15^ M when Pt NPs/Eastman-AQ55D/ [Ru(bpy)_3_]^2+^ is used to fabricate the sensor. The linear response range is broad, around 10^4^ - 10^5^ M. Meanwhile, other ECL systems have also been exploited in sensor development, including [Ru(bpy)_3_]^+/3+^ annihilation, the luminol/H_2_O_2_, and quantum dot-based ECL. These and other configurations compared in Table 1 are described below.

### 2.1. Thin Films

Unlike luminol which is consumed, [Ru(bpy)_3_]^2+^ can be regenerated. Thus, it can be immobilized onto an electrode surface and reused in a thin-film sensor platform [4]. A [Ru(bpy)_3_]^2+^-containing thin-film sensor produces less heavy metal waste, requires a less complicated pumping system, and reduces costs compared with a solution-based sensing method [5]. Nafion is an effective ion exchanger for [Ru(bpy)_3_]^2+^ and has been used for [Ru(bpy)_3_]^2+^ immobilization [6]. However, a pure Nafion film sensor is far from ideal. [Ru(bpy)_3_]^2+^ leaching and partitioning into hydrophobic regions of the film, slow mass transfer of the analyte, and the instability of the films in organic solvents are drawbacks to be overcome with these sensors [4,7]. Nevertheless, [Ru(bpy)_3_]^2+^ incorporation into Nafion has seen some success because of both ion-exchange and hydrophobic interactions [4]. Langmuir-Blogdett films and self-assembled monolayers have largely failed in the case of [Ru(bpy)_3_]^2+^-based sensors because the high potentials required to produce an ECL signal are detrimental to these films [8]. So far, two effective ECL films are sol-gel film and polymer films.

#### 2.1.1. Sol-gels

Many recent successes with thin-film ECL sensor platforms have been achieved with sol-gels. Pastore *et al.* successfully trapped [Ru(bpy)_3_]^2+^ in a silica glass thin film on a fluoride-doped tin oxide electrode (K-glass electrode). The film was transparent, chemically inert, physically rigid, and stable for over 1,000 experiments [5]. Dong and co-workers reported a composite film of Eastman-AQ55D cation-exchange polymer and silica sol-gel as an effective means for direct [Ru(bpy)_3_]^2+^ immobilization. Eastman-AQ55D has excellent ion-exchange properties and is more hydrophobic than Nafion resulting in better retention of the hydrophobic [Ru(bpy)_3_]^2+^ ion and thus a more stable sensor [7].

Other sensing platforms have combined sol-gels and Nafion films. For example, Lee *et al.* have reported sol-gel-derived TiO_2_-Nafion composite films for effective immobilization of Ru(bpy)_3_^2+^ at an electrode surface. This ECL sensor showed improved ECL sensitivity as compared with ECL sensors based on pure Nafion films. The reason was ascribed to larger pores and thus faster diffusion of analytes into the film [4]. Thereafter, the same group developed mesoporous films of V_2_O_5_/Nafion composites as effective materials to immobilize [Ru(bpy)_3_]^2+^. It was found that the composite film with 80% Nafion content had the largest pore diameter (4.19 nm) and highest ECL yields with tripropylamine (TPA). The greater sensitivity was the result of not only the large pores that offered rapid analyte diffusion, but also the relatively high conductivity of V_2_O_5_. Based on these advantages, this ECL sensor exhibited an approximately two orders of magnitude higher ECL response and one order of magnitude lower detection limit for TPA (10 nM) compared with ECL sensors based on SiO_2_/Nafion and TiO_2_/Nafion [9].

The growing trend toward use of nanotechnology has also made its way into the formation of thin-film sensing platforms. Xu and coworkers have created three-dimensional sensing platforms by depositing gold on silica nanoparticle templates. Using HF to dissolve the nanoparticles, a macroporous gold structure with high surface area and excellent conductivity was left behind, onto which a ZrO_2_/Nafion film containing [Ru(bpy)_3_]^2+^ was deposited. Compared with flat surfaces, increased [Ru(bpy)_3_]^2+^ loading was achieved with this configuration thereby yielding high sensitivity [8].

#### 2.1.2. Metallopolymers

**Figure 2.**
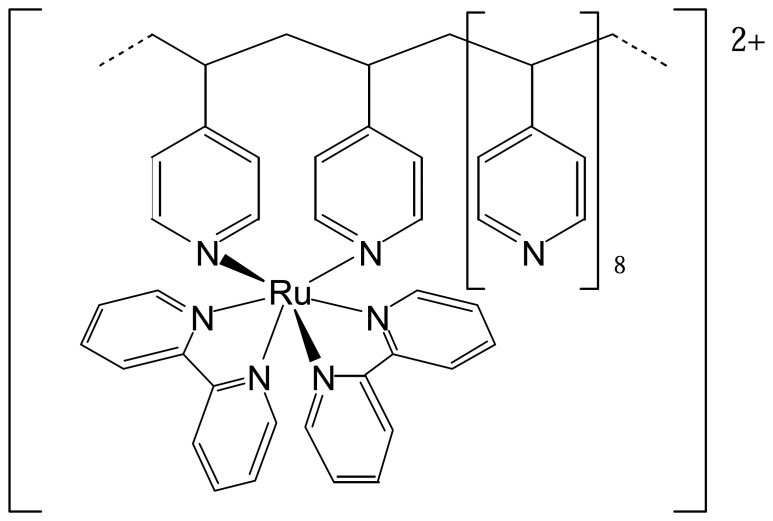
[Ru(bpy)_2_(PVP)_10_]^2+^, PVP = poly(4-vinylpyridine).

Metallopolymers, or polymers containing metal cores, provide an additional method for ECL reagent immobilization since they can be directly deposited onto an electrode. Polymers which contain a Ru^2+^ core (e.g., Figure 2) can produce ECL by a mechanism similar to [Ru(bpy)_3_]^2+^. The polymer matrix has been shown to give a four-fold improvement in ECL efficiency compared with that obtained for [Ru(bpy)_2_(PVP)_10_]^2+^ dissolved in solution, due mainly to protection from oxygen quenching and competing side reactions. Forster *et al.* demonstrated ECL with the metallopolymer in Figure 2 based on both annihilation and co-reactant mechanisms [10].

In an additional report by Lee *et al.*, [Ru(bpy)_3_]^2+^ was modified with –Si(OMe)_3_ groups on each ligand (Figure 3). After hydrolysis of the silicate groups, a thin, porous polymer film was immobilized on an ITO (indium-tin oxide) electrode. The reason for choosing ITO as the electrode material was that the hydroxyl groups on its surface would also bound with the modified [Ru(bpy)_3_]^2+^ through –Si—O— bonds. The film was very stable, even after long-term exposure to acetonitrile [11].

### 2.2. Electrode Arrays

The benefit of having an array of microelectrodes rather than a single electrode is that many data can be collected simultaneously, thus arrays can reduce interferences through data processing [12]. Furthermore, they can allow for simultaneously determination of multiple analytes [13].

In the case of the electrochemical signal produced by an array, individual electrodes are indistinguishable. In contrast, the ECL signals for individual electrodes can be read separately with a CCD camera or an optical fiber bundle [14]. Sojic *et al.* created a microelectrode array by coating conical glass cores with ITO then insulating the surface except for the apex of the tips with electrophoretic paint. ECL signals from the submicron sensors for both [Ru(bpy)_3_]^2+^/TPA [14] and luminol/H_2_O_2_[12] were individually read with optical fibers. Although the electrodes could be read individually they could not be controlled individually, and thus only one analyte could be detected at a time [12, 14]. Alternatively, Marquette and coworkers modified glassy-carbon foil electrodes in order to detect multiple analytes simultaneously. The enzymatic production of H_2_O_2_ was detected for all analytes, but at different areas of the electrode [15]. Oxidase enzymes were spotted on the foil electrode and non-covalently immobilized. Luminol ECL was used to detect H_2_O_2_ that was enzymatically produced in the presence of the respective substrates [13, 16].

### 2.3. Microfluidics

The relatively new field of microfluidics has advantages of flexible cell designs, low cost, miniaturization and automation. However, the most sensitive detectors for these devices, such as laser-induced fluorescence (LIF) and mass spectroscopy (MS), are bulky and expensive. Electrochemical detection systems are generally less expensive and more spatially compact, but they are not as sensitive as the above mentioned detectors. ECL sensing platforms should play an important role in this area because they not only rival the sensitivity of more expensive LIF and MS detection systems, but they have the same size advantages of electrochemical systems [17]. A recent example of ECL detection in microfluidics was reported by Pittet *et al.* who detected hydrogen peroxide with luminol ECL. Printed circuit board electrodes were used in this example, which can be fabricated inexpensively in large-scale production [3].

Crooks and coworkers have also developed a two-electrode microfluidic device where targets are detected electrochemically at the cathode (Equation 12), and are reported via ECL at the anode (Equation 13). By this method, it is not necessary for the target analyte to have the capacity to react with [Ru(bpy)_3_]^3+^ to produce ECL, it need only be redox active.

(12) $$\text{Cathode}:\text{O}+{\text{e}}^{-}\to \text{R}$$

(13) $$\text{Anode}:{\left[\text{Ru}{\left(\text{bpy}\right)}_{3}\right]}^{2+}\to {\left[\text{Ru}{\left(\text{bpy}\right)}_{3}\right]}^{3+}+{\text{e}}^{-}$$

Since the two working electrodes are in electrochemical communication and a charge balance must be maintained, the reaction at the cathode shown in Equation 12 gives rise to the anodic reaction (Equation 13). TPA, present in solution, is able to react with [Ru(bpy)_3_]^3+^ to produce ECL. The two channels are connected by an outlet. However, due to laminar flow, there is no bulk mixing [17].

The same group also reported a three-channel system where both the target analyte detection and the ECL reporting occur at the anode. Therefore, oxidation of the target competes with oxidation of [Ru(bpy)_3_]^2+^, and its presence causes a decrease in ECL signal [18].

### 2.4. Nanomaterials used in recent ECL sensors

Nanotechnology is defined as the fabrication of structures or devices on the atomic or molecular scale, but also includes devices that are less than 100 nm in at least one dimension [19]. Nanomaterials encompass a broad range of materials, including nanoparticles, nanorods, nanocrystals, nanowires and nanotubes of virtually any chemical composition. Due to the small size, usually nanomaterials experience light scattering when used in optical measurements. However, this drawback can be completely eliminated in ECL determination since no radiation source is needed in ECL. Thus, the advantages of both nanomaterials and ECL can be fully exploited when an ECL sensor is made of nanomaterials. Nanomaterials can enhance sensitivity by increasing the surface area [20] or enhancing conductivity [21] of a sensor platform. For example, porous nanoparticles were used to sequester high concentrations molecules of the ECL-producing agent, resulting in labels with greater luminescence [22]. Quantum dots are nanoparticles that have their own intrinsic ECL-producing ability [23]. This section explores some of the recent applications of several types of nanoparticles, as well as carbon nanotubes, in fabrication of ECL sensors.

#### 2.4.1. Nanoparticles

##### Gold Nanoparticles

Gold nanoparticles have found use in ECL sensors due to their biocompatibility [24], high electrical conductivity, and the ease by which they can be self assembled through formation of Au-thiol and Au-amine bonds [25]. Cui *et al.* created a sensor for determination of H_2_O_2_ using immobilized luminol. The challenges of luminol immobilization are that it often causes a decrease in luminescence efficiency and that the luminol supply is exhausted by irreversible ECL reaction. It was found that luminol can be used to reduce chloroauric acid (HAuCl_4_) to form gold nanoparticles. The resultant gold nanoparticles with a residual coating of luminol and 3-amionphthalate (AP^2-^) were weakly attached to the luminol through covalent Au-N bonds. By immersing the electrode in a luminol solution, the AP^2-^ could be replaced with luminol through an ion-exchange mechanism. These gold nanoparticles were immobilized at a gold electrode and, by applying a potential pulse rather than a constant potential, the immobilized luminol supply was exhausted at a slower rate. In fact, after 600 pulses and five hours, the signal had maintained 80% of its intensity [26].

Dong and coworkers fabricated an alcohol biosensor by immobilizing gold nanoparticle/[Ru(bpy)_3_]^2+^ aggregates with alcohol dehydrogenase (ADH). [Ru(bpy)_3_]^2+^ often causes enzymes to lose activity either due to its hydrophobic nature or its positive charge which can effect enzyme orientation. Here, the gold nanoparticles were effective in separating the ADH from the [Ru(bpy)_3_]^2+^ preventing this effect [25].

##### Platinum Nanoparticles

Like gold nanoparticles, platinum nanoparticles have also been used in electrochemical sensors due to their high electrical conductivity and electroactivity. Wang and coworkers have demonstrated their use in an ECL sensor based on an Eastman AQ55D cation-exchange polymer film containing [Ru(bpy)_3_]^2+^. When Pt nanoparticles were also present in the film, the sensor exhibited faster electron transfer, higher ECL intensity, and a shorter Equilibrium time than when they were absent. These factors resulted in higher sensitivity when TPA was the analyte and an extremely low detection limit of 10^-15^ M [21].

##### Magnetic Nanoparticles

Magnetic nanoparticles made of Fe_3_O_4_ have the advantage of being easily separated and immobilized. By coating these particles with [Ru(bpy)_3_]^2+^, ECL sensors have been made [26,27]. Lee *et al.* have coated magnetic nanoparticles with Nafion in which they were able to incorporate [Ru(bpy)_3_]^2+^ after immobilization onto the electrode. The uptake of [Ru(bpy)_3_]^2+^ at the Nafion-coated nanoparticles was much faster than into Nafion alone, probably due to the accessibility of the SO_3_^-^ sites. The resulting sensor was stable. Its signal decreased to 80% the original after one month of storage [27]. Dong and coworkers had a very similar set-up, except they used a silica shell for [Ru(bpy)_3_]^2+^ incorporation rather than the Nafion coating [26].

##### Silica Nanoparticles

Silica nanoparticles are versatile in the number of functional groups with which they can be modified and the variety of species they can sequester. These species includes ECL producing agents such as [Ru(bpy)_3_]^2+^ ions [28,29] and luminol [30].

However, even without doping [Ru(bpy)_3_]^2+^, silica nanoparticles can still be useful for ECL sensing by increasing the surface area of the sensor platform and providing the space for co-reactants to diffuse. Dong *et al.* created a sensor in which [Ru(bpy)_3_]^2+^ and silica nanoparticles were immobilized together using the layer-by-layer method. The ECL signal was an order of magnitude higher than previous sensor films [20].

Dong and coworkers also immobilized [Ru(bpy)_3_]^2+^-doped silica nanoparticles on an electrode in a chitosan biopolymer film. The sensor was especially stable because of the electrostatic interactions between the [Ru(bpy)_3_]^2+^ and the negatively charged silica, which prevented leaching. Additional stability was gained due to the hydrogen bonding between the chitosan and the nanoparticles. Eighty percent of the original ECL signal remained after 80 days [28]. Later, the same group reported a similar sensor that employs carbon nanotubes to not only immobilize the [Ru(bpy)_3_]^2+^-doped silica nanoparticles, but also accelerate electron transfer. Because silica is a rather insulating material, electron transfer assistance was invaluable. This sensor was also very stable, as it remained at 85% its original signal after 20 days [29].

[Ru(bpy)_3_]^2+^-doped silica nanoparticles have also been used as labels in bioassays [22,31], which will be discussed in Section 3.

##### Quantum Dots

Many types of semiconductor nanoparticles-also called quantum dots (QDs)-can undergo redox chemistry. This electron or hole injection can produce radiation emissions if the resulting charged states are sufficiently stable to survive until colliding with an oppositely charged specie in an annihilation reaction (Equations 14 and 15) [23]. This is the case for silicon nanocrystals [23] and CdTe quantum dots [32] as reported by Bard and coworkers, although the precise mechanism is still unknown.

(14) $${\text{QD}}^{+•}+{\text{QD}}^{-•}\to {\text{QD}}^{*}+\text{QD}$$

(15) $${\text{QD}}^{*}\to \text{QD}+hv$$

In addition to the annihilation mechanism, co-reactants such as hydrogen peroxide, can also produce an ECL signal. Thus, two ECL peaks are often observed [33]. Since ECL of quantum dots has only recently been explored, it still has many disadvantages. For instance, a high negative potential is required before ECL is produced [34], and the intensity is not at the level of [Ru(bpy)_3_]^2+^ or luminol ECL [35]. It is expected that these issues will be addressed and remedied in forthcoming research papers.

An early example of a sensor based on quantum dot ECL was given by Ju and coworkers who immobilized CdSe nanoparticles in a paraffin film on a graphite electrode. By scanning to negative potentials, the quantum dots produced ECL in the presence of H_2_O_2_ (Equations 16-18) with a detection limit of 0.1 mM [36]. Later, they showed that thiols had the ability to quench the signal by reacting with hydroxyl radicals (Equations 19 and 20), a characteristic which could be exploited in a sensor [37].

(16) $$\text{QD}+{\text{e}}^{-}\to {\text{QD}}^{-•}$$

(17) $${\text{QD}}^{-•}+{\text{H}}_{2}{\text{O}}_{2}\to \text{QD}+{\text{OH}}^{•}+{\text{OH}}^{-}$$

(18) $${\text{QD}}^{-•}+{\text{OH}}^{•}\to {\text{OH}}^{-}+{\text{QD}}^{*}$$

(19) $${\text{OH}}^{•}+\text{RSH}\to {\text{RS}}^{•}+{\text{H}}_{2}\text{O}$$

(20) $$2{\text{RS}}^{•}\to \text{RS}—\text{SR}$$

Chen and coworkers have produced a similar sensor using carbon paste for immobilization and using CdS nanotubes as the nanoscale semiconductor material. The CdS nanotubes were 100 – 140 nm in diameter, with nanocrystals ∼7 nm in diameter, and exhibited properties similar to quantum dots. ECL signal s were observed for both the annihilation and co-reactant mechanisms and the sensor was used to detect S_2_O_8_^2-^, H_2_O_2_, and dissolved oxygen [35].

Later, Chen *et al.* developed a sensor for H_2_O_2_ based on a multilayer film on a GCE consisting of CdS quantum dots and hemoglobin. The heme prosthetic group in the hemoglobin acted as a peroxidase and catalytically reduced H_2_O_2_. In addition, the hemoglobin seemed to stabilize the quantum dots. In the absence of hemoglobin, the ECL signal vanished after 30 cycles, whereas it remained stable after 30 cycles in the presence of hemoglobin [34].

Zhu and coworkers found that carbon nanotubes were able to address some of the disadvantages of QD ECL. When CdS quantum dots and CNTs were immobilized together, the ECL signal was 5-fold greater when H_2_O_2_ was the co-reactant and the potential at which it was initiated shifted anodically from -1.15 to -0.85 V. The CNTs were believed to decrease the potential barrier for QD reduction (Equation 5) in addition to providing a more porous structure for faster H_2_O_2_ diffusion [33].

#### 2.4.2. Carbon Nanotubes

The conducting properties of carbon nanotubes (CNTs) can be extremely helpful in accelerating electron transfer for electrochemical and ECL-based sensors. CNTs are graphene sheets cylindrically rolled to nanometer-diameter tubes. When CNTs are incorporated into a sensor platform, they can act as conducting pathways between the lumophores and the electrode. In addition, they increase surface area and porosity of the platform, making co-reactant diffusion faster [6]. The benefits of CNTs to quantum dot ECL have already been mentioned in the previous section.

Dong *et al.* developed a composite film of Nafion and carbon nanotubes, into which [Ru(bpy)_3_]^2+^ was incorporated. When the film was assessed with TPA, it was found that the ECL signal was two orders of magnitude greater than when silica was used in the place of CNTs. There was a three-orders-of-magnitude difference when only Nafion was used for the film [6]. Wang and coworkers produced a sensing platform using partial sulfonation polystyrene and CNTs. Similarly, the carbon nanotubes gave an increase of three orders of magnitude in the ECL signal than with the polymer alone [38]. Lee and coworkers developed a film containing CNTs, Nafion and titania sol-gel with [Ru(bpy)_3_]^2+^. This film was extremely stable, having no signal loss for four months [39]. Chen *et al.* developed a organically modified silicate film to immobilize poly(*p*-styrenesulfonate)-coated multi-walled CNTs. [Ru(bpy)_3_]^2+^ was incorporated into this film through ion exchange [40].

Although the recent focus seems to be on [Ru(bpy)_3_]^2+^ ECL, luminol can also benefit from CNTs. A CNT paste electrode was developed and infused with glucose oxidase (GOx) for a glucose sensor by Chen and coworkers. The luminol was injected in the solution phase and was able to react with H_2_O_2_ which was produced in the catalysis of glucose by GOx [41].

## 3. Sensing Applications of ECL

### 3.1. Label-Free Sensors

#### 3.1.1. Luminol/Hydrogen Peroxide

Luminol is useful for H_2_O_2_ determinations since reactions between luminol and H_2_O_2_ can produce ECL. H_2_O_2_ is also a product in many enzymatic reactions where substrates are oxidized by O_2_. A prototypical example is the oxidation of glucose by the oxidoreductase enzyme, glucose oxidase (GOx) (Equation 21). Thus H_2_O_2_ determinations can be an indirect method for the determination of biological compounds. Such is the case in the reports of Marquette *et al.*, which described sensor arrays for simultaneous determinations of glucose, lactate, choline, glutamate, lysine and urate [13]. Oxidase enzymes were immobilized on the electrode surface where they could catalyze the production of H_2_O_2_, which would in turn react with luminol and produce ECL. Detection limits for the biological compounds were typically in the micromolar range [12, 14, 15].

(21) $$\beta -D-\text{glucose}+{\text{O}}_{2}\overset{\text{GOx}}{\to }\text{gluconicacid}+{\text{H}}_{2}{\text{O}}_{2}$$

Zhang and coworkers reported an example of luminol ECL produced by a galvanic cell to detect H_2_O_2_. Since galvanic cells require no external power source, cost and size of the sensor could be significantly reduced. A Cu/Zn alloy produced the galvanic cells *via* the corrosion effect. In alkaline solution, the zinc dissolved from the alloy anodically, producing a potential of +1.1 V which is sufficient for ECL generation with luminol. The presence of H_2_O_2_ caused the chemiluminescence signal to increase. The detection limit for hydrogen peroxide was 0.3 μM [42].

**Figure 4.**
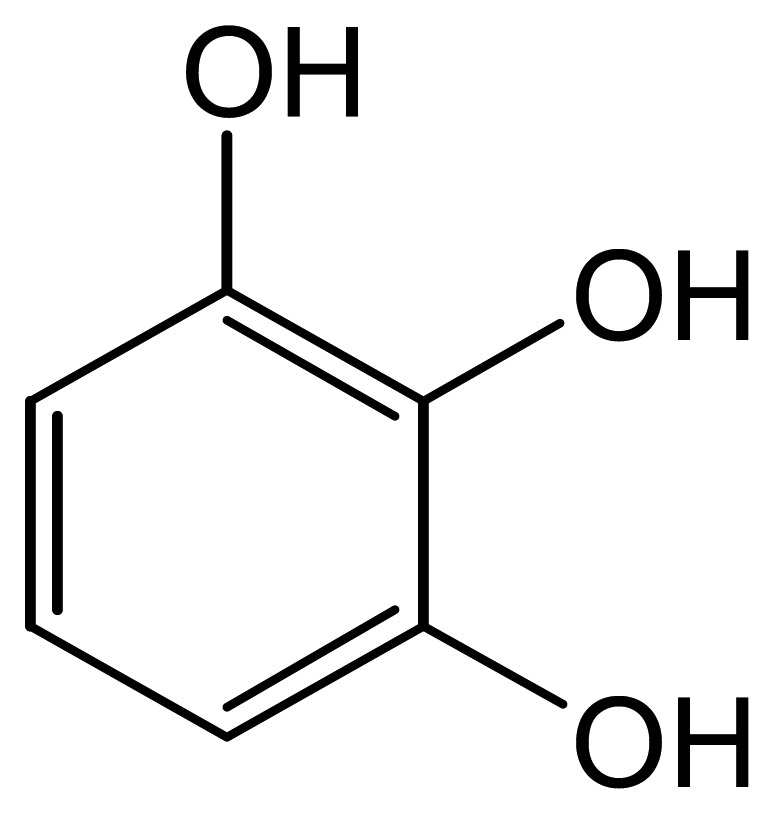
Pyrogallol.

Zhang and Zheng produced a sensor for pyrogallol (Py, Figure 4) that used luminol-doped silica nanoparticles to produce an ECL signal. The nanoparticles were immobilized at a graphite electrode in a chitosan film through which pyrogallol could diffuse and was electrochemically oxidized (Equation 22). The resulting pyrogallol radical could reduce oxygen to a anion radical which in turn reduces luminol to form AP^2-^*, the ECL-producing product (Equations 23 and 24). ECL was therefore dependent on pyrogallol concentration. The reported detection limit was 1.0 nM [30].

(22) $$\text{Py}\to {\text{Py}}^{•}+{\text{e}}^{-}+{\text{H}}^{+}$$

(23) $${\text{Py}}^{•}{+\text{O}}_{2}\to {{\text{O}}_{2}}^{•-}+{\text{Py}}_{\text{ox}}$$

(24) $$2{{\text{O}}_{2}}^{•-}+\text{luminol}\to {\text{AP}}^{2-*}+{\text{N}}_{2}$$

#### 3.1.2. [Ru(bpy)_3_]^2+^/Amines

The ability of [Ru(bpy)_3_]^3+^ to react with amine radicals to produce ECL makes it useful in detecting a variety of important analytes. Among them is guanine (G), the DNA base (Equations 25-27) [43]. Furthermore, [Ru(bpy)_3_]^3+^ can more easily react with single stranded (ss) DNA compared with double stranded (ds). Reported rates for the reaction shown in Equation 25 were 9×10^3^ M^-1^s^-1^ for ds calf thymus DNA and 2×10^5^ M^-1^s^-1^ for ss calf thymus DNA. This characteristic makes the ECL method particularly useful in detecting base mismatches [43]. Forster and coworkers used the metallopolymer described in Section 2 to detect chemical damage to ds-DNA. DNA and the metallopolymer were deposited alternately using the layer-by-layer method. The films were then incubated with styrene oxide to cause chemical damage. The group was able to detect 0.1% damage, or one base in 1000 [43].

Wang *et al.* used a previously reported CNT and Nafion composite film [38] in which [Ru(bpy)_3_]^2+^ was incorporated in order to detect DNA. DNA was non-specifically bound to the film, and again, ss-DNA gave a more intense ECL signal. By exposing the films to a boiling water bath for five minutes, native ds-DNA was denatured and the ECL signals produced before and after this treatment could be compared. In this way, single-base mismatch could be detected. In addition, the group found that adenine also was able to contribute to the ECL signal [44].

Chen and coworkers used their organically modified silica film with PSS-coated CNTs [40], which was discussed in Section 2.4.2, to detect herring sperm DNA. The ability of guanine to oxidize (to G_ox_) and behave as a co-reactant in [Ru(bpy)_3_]^2+^ ECL (Equations 25-27) was also exploited in this flow-injection analysis sensor. A modest detection limit of 0.2 mg L^-1^ was achieved [40].

(25) $${\left[\text{Ru}{\left(\text{bpy}\right)}_{3}\right]}^{2+}\to {\left[\text{Ru}{\left(\text{bpy}\right)}_{3}\right]}^{3+}+{\text{e}}^{-}$$

(26) $${\left[\text{Ru}{\left(\text{bpy}\right)}_{3}\right]}^{3+}+\text{DNA}(\text{G})\to {\left[\text{Ru}{\left(\text{bpy}\right)}_{3}\right]}^{2+}+{\text{DNA}}^{•+}({\text{G}}_{\text{ox}})$$

(27) $${\text{DNA}}^{•+}({\text{G}}_{\text{ox}})+{\left[\text{Ru}(\text{bpy})3\right]}^{3+}\to {\text{DNA}}^{2+}({\text{G}}_{2\text{OX}})+{\left[{\text{Ru}(\text{bpy})}_{3}\right]}^{2+*}$$

Lee and coworkers have used the TiO_2_/Nafion composite sensor [3] discussed in Section 2.1.1 for determinations of phenothiazine derivatives in urine samples after HPLC separation [45]. Phenothiazines are a group of compounds often used in anti-depressant drugs. Therefore, monitoring them in bodily fluids is important in order to minimize toxicity risk. They contain aliphatic tertiary amines, which allow their detection with [Ru(bpy)_3_]^2+^-based ECL [45].

**Figure 5.**
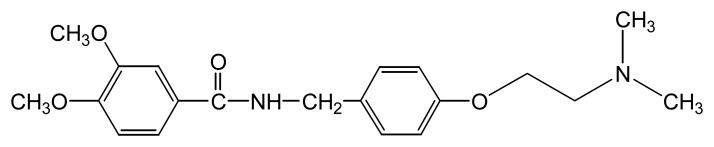
Itopride.

Zhang and coworkers have used a sensor composed of [Ru(bpy)_3_]^2+^-doped silica nanoparticles immobilized at an electrode surface in a chitosan film to detect itopride (*N*-[4-[2-(dimethylamino)-ethoxy]benzyl]-3,4-dimethoxybenzamide hydrochloride) (Figure 5) [46]. Their sensor was similar to the one reported by Dong *et al.* for TPA determinations [7]. Itopride is a new drug prescribed for a variety of gastrointestinal symptoms, thus its detection in biological samples is important for optimization of dosages. Since it contains a tertiary amine, it is a candidate for an ECL sensor. A detection limit of 3 ng mL^-1^ was reported. An analysis of human serum samples was also performed and showed good percent recoveries [46].

### 3.2. ECL Labels

#### 3.2.1. Molecular Labels

Kuwabara and coworkers reported that the ECL-producing ruthenium complex [Ru(phen)_3_]^2+^ was able to bind to the major groove of DNA. The result was that DNA added to a solution containing [Ru(phen)_3_]^2+^ and oxalate caused a decrease in ECL signal. The ruthenium complex was therefore used as a probe to determine the binding mode of certain anti-cancer drugs. Evaluating the binding modes of drugs is an important step towards understanding the drug mechanism. If the drugs also preferred binding to the major groove, an increasing drug concentration would have a coinciding increasing ECL signal due to liberation of [Ru(phen)_3_]^2+^. On the other hand, if the drug only bound to the minor groove, concentration would have no effect [47].

Modified [Ru(bpy)_3_]^2+^ has been used as a molecular label in DNA and C-reactive protein (CRP) determinations by Miao and Bard. For DNA, a thiol-modified capture strands complimentary to the target were self assembled onto a gold electrode. The targets were labeled with [Ru(bpy)_3_]^2+^. The ECL signal resulting from a TPA radical produced at strong potentials was then proportional to the target. Oxidation of the SAM was not addressed as a major problem, however, it was noted that the maximum ECL signal appeared at +0.95 V which is less positive than with free [Ru(bpy)_3_]^2+^. A similar method for CRP determination was reported in the same article. Biotinylated anti-CRP was immobilized on an avidin surface bound to a gold-thiol SAM in a sandwich-type assay. An anti-CRP probe was labeled with [Ru(bpy)_3_]^2+^, which gave an ECL signal proportional to the CRP concentration [48].

Zhang and coworkers reported cocaine determinations a [Ru(bpy)_3_]^2+^-modified aptamer which was immobilized to a gold electrode via the self-assembly method. The [Ru(bpy)_3_]^2+^ molecular probe at the end of a random coil did not give a strong ECL signal because of it distance from the electrode. However, after the binding of cocaine to the aptamer, the [Ru(bpy)_3_]^2+^ probe was in close proximity with the electrode, therefore giving a strong ECL signal in the presence of TPA when a potential of +0.8 V was applied. It was also noted that potentials more positive than +1.1 V gave poor reproducibility due to the oxidation of the thiol SAM. The detection limit for this method was reported at 1 nM cocaine [49].

Blum and coworkers used a derivative of luminol, *N*-(4-aminobutyl)-*N*-ethylisoluminol (ABEI) as a molecular probe for DNA determinations. Their capture DNA was modified with pyrrole on one end for the purposes of immobilization. A pyrrole film was electrochemically deposited in the electrode with the pyrrole-modified DNA. The probe DNA was modified with biotin, as was the ABEI, therefore they could be connected through an avidin bridge. It was shown that a complimentary probe gave a higher ECL signal in the presence of 1.3 mM H_2_O_2_ than a non-complimentary probe in a brief demonstration [50].

#### 3.2.2. Micro- and Nanoparticle Labels

Micro- and nanoparticles that contain ECL-producing agents, primarily [Ru(bpy)_3_]^2+^, can also be used as labels in sensors. Because they contain [Ru(bpy)_3_]^2+^ ions, the signals from these types of sensors can be greatly enhanced over molecular labels.

**Figure 6.**
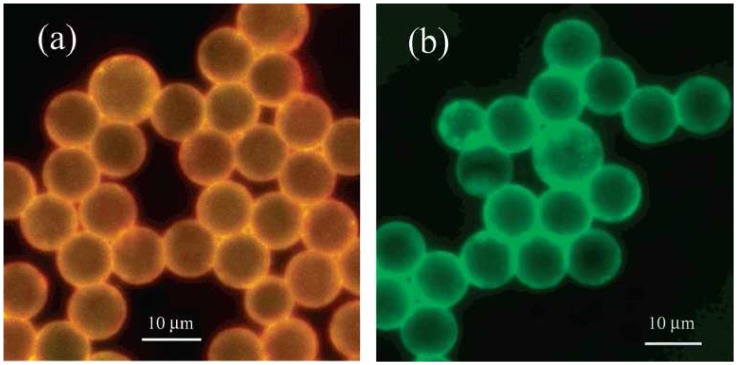
Fluorescent images (*λ*_ex_ 490 nm, exposure time 30 s) of carboxylate polystyrene beads: (a) after entrapment of Ru(bpy)_3_[B(C_6_F_5_)_4_]_2_ and (b) after covalent binding of avidin to the surface. Reproduced with permission of ACS [51].

In an early example, Miao and Bard reported DNA [51] and CRP [52] determination with polystyrene microspheres (10 mm) containing the water-insoluble specie Ru(bpy)_3_[B(C_6_F_5_)_4_]_2_ (Figure 6). These reports are similar to the molecular probe example discussed in section 3.2.1 [48]. Each microsphere contained approximately 7.5×10^9^ molecules and was attached to a target DNA strand. The complimentary probe DNA was attached to a magnetic bead which could hybridize with the labeled target. The hybridized target DNA could then be magnetically separated from the single stranded targets [51]. The CRP sensor was a sandwich-type assay in which anti-CRP was attached to the polystyrene mircospheres as well and the magnetic beads. When these both bound to the CRP they could be magnetically separated to collect the polystyrene beads [52]. The polystyrene microspheres were then dissolved in acetonitrile and [Ru(bpy)_3_]^2+^ was determined by ECL with TPA as the co-reactant. Detection limits of 1.0 fM DNA were achieved [51] and 10 mg L^-1^ CRP [52]. Zhan and Bard also reported a CRP sensor that used [Ru(bpy)_3_]^2+^-containing liposomes (100 nM) as labels. The advantage of using liposomes rather than polystyrene microspheres was than the [Ru(bpy)_3_]^2+^ could be released with sodium chloride and a surfactant, which are more benign than acetonitrile. However, the detection limit was an order of magnitude higher at 100 μg L^-1^[53].

**Figure 7.**
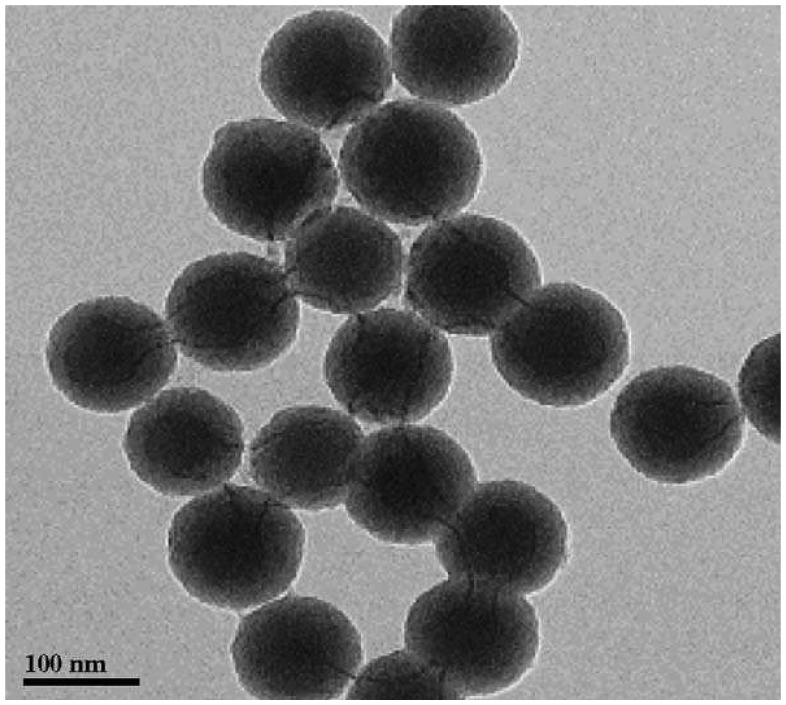
TEM image of Ru(bpy)_3_^2+^-doped silica nanoparticles. Reproduced with permission of Elsiever [22].

Fang and coworkers reportedly used [Ru(bpy)_3_]^2+^-doped silica nanoparticles (Figure 7) as labels for DNA hybridization determinations [22]. Capture DNA was immobilized at a polypyrrole modified platinum electrode, similarly to the method described in the previous section [50]. The silica nanoparticles were modified with complimentary target DNA which could hybridize with the DNA on the electrode surface. Unlike the method reported by Miao and Bard [51], the [Ru(bpy)_3_]^2+^ ions were not released. However, the co-reactant, oxalic acid, could penetrate the nanoparticles and allowed for the production of ECL proportional to the amount of target DNA. A detection limit of 0.1 pM was reported [22].

The same group reported a thrombin aptasensor using [Ru(bpy)_3_]^2+^-doped silica nanoparticles as labels. In this example, aptamers were immobilized at a gold electrode by Au-S bonds. Hybridization of complimentary DNA with the silica nanoparticle labels resulted in an ECL signal in the presence of TPA and positive potentials. With the addition of thrombin, the nanoparticle-labeled DNA was displaced, thus decreasing the ECL signal. The signal decrease was proportional to the thrombin concentration. Despite the method for aptamer immobilization (SAM), there was no report of monolayer instability, even though potentials of +1.2 V were applied. The reported detection limit was 1.0 fM [31].

## Conclusions

While [Ru(bpy)_3_]^2+^ and luminol continue to be the preferred lumophores used in most recent ECL sensors, the use of micro- and nanobased materials as supports has significantly enhanced the performance of these species in sensing applications. In the case of inherently luminescent quantum dots, the application of new nanomaterials has also provided an entirely different sensing paradigm. With the present, rapid development of many other new nanomaterials and microfluidic analysis systems, it is clear that ECL sensors will continue to benefit from incorporation of these reduced-scale constructs and play an important role in future analytical applications.

## Figures and Tables

**Fgure 3.**
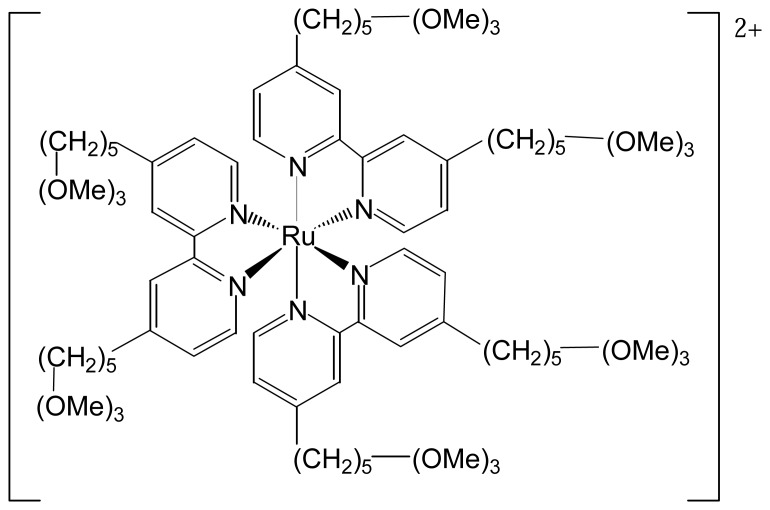
A Si(OMe)_3_-modified [Ru(bpy)_3_]^2+^ used for immobilization on ITO electrodes.

**Table 1.** Characteristics of ECL sensors based on the [Ru(bpy)_3_]^2+^/TPA system.

**Sensor Platform**	**LOD, M**	**Linear Range, M**	**RSD, %**	**Ref.**
Silica/Eastman-AQ55D/ [Ru(bpy)_3_]^2+^	1 × 10^-7^	2 × 10^-5^ – 1 × 10^-3^	1.9	5
TiO_2_/Nafion/[Ru(bpy)_3_]^2+^	1 × 10^-7^	1 × 10^-7^ – 1 × 10^-3^	3.9	4
V_2_O_5_/Nafion/[Ru(bpy)_3_]^2+^	1 × 10^-8^	5 × 10^-8^ – 1 × 10^-3^	2.5	9
ZrO_2_/Nafion (on 3D Au structure)/ [Ru(bpy)_3_]^2+^	5 × 10^-10^	1 × 10^-9^ – 1 × 10^-5^	0.74	8
Pt NPs/Eastman-AQ55D / [Ru(bpy)_3_]^2+^	1 × 10^-15^	–	0.6	21
Fe_3_O_4_ NPs/Nafion/[Ru(bpy)_3_]^2+^	5 × 10^-8^	1 × 10^-7^ – 1 × 10^-3^	3.9	27
Fe_3_O_4_ NPs/Silica/[Ru(bpy)_3_]^2+^	6.5 × 10^-9^	6.9 × 10^-8^ – 7.3 × 10^-4^	0.5	26
SNPs/[Ru(bpy)_3_]^2+^	1 × 10^-8^	2.6 × 10^-8^ – 1.3 × 10^-3^	5.2	20
[Ru(bpy)_3_]^2+^ SNPs/chitosan	2.8 × 10^-9^	8.5 × 10^-9^ – 8.1 × 10^-5^	–	28
[Ru(bpy)_3_]^2+^ SNPs/CNTs	2.8 × 10^-9^	8.5 × 10^-9^ – 7.9 × 10^-4^	–	29
Nafion/CNTs/[Ru(bpy)_3_]^2+^	1 × 10^-9^	3 × 10^-9^ – 1 × 10^-4^	<10	6
PSP/CNTs/[Ru(bpy)_3_]^2+^	6 × 10^-9^	–	–	38
Nafion/CNTs/TiO_2_/[Ru(bpy)_3_]^2+^	1 × 10^-8^	5 × 10^-8^ – 1 × 10^-3^	<4	39
